# Successful Treatment of Autoimmune Hemolytic Anemia with Steroid, IVIg, and Plasmapheresis in a Haploidentical Transplant Recipient

**DOI:** 10.5505/tjh.2012.78055

**Published:** 2012-06-15

**Authors:** Burak Uz, Evren Özdemir, Salih Aksu, Tülay Karaağaç Akyol, Roy Jones

**Affiliations:** 1 Hacettepe University, School of Medicine, Department of Hematology, Ankara, Turkey; 2 Hacettepe University, School of Medicine, Department of Oncology, Ankara, Turkey; 3 Hacettepe University, School of Medicine, Blood Bank and Apheresis Unit, Ankara, Turkey; 4 The University of Texas, MD Anderson Cancer Center, Department of Stem Cell Transplantation, Houston, Texas, USA

## TO THE EDITOR

Autoimmune hemolytic anemia (AIHA) is a rare, but clinically significant complication following hematopoietic stem cell transplantation (HSCT). It is characterized by hemolysis due to antibodies produced by the donor’s immune system against donor red cell antigens. The 3-year cumulative incidence of AIHA is 4.44% in adults; however, 75% of patients develop AIHA during the first post-HSCT year [1]. AIHA after allogeneic HSCT has been associated with a variety of conditions, including chronic graft-versus-host disease (GVHD) [2], T-cell depletion [3], and unrelated donor transplants [4]. HSCT from unrelated donors and the development of chronic extensive GVHD were the only independent factors associated with AIHA [1]; however, the incidence of and risk factors for AIHA, as well as its prognosis and response to treatment remain unclear. 

A 56-year-old male was diagnosed with high-risk acute myelogenous leukemia in April 2008. He was administered 8 cycles of chemotherapy, including idarubicin, cytosine arabinoside (ara-C), and sorafenib, and achieved complete remission. He remained in remission for 17 months, and then relapsed. He was treated with ara-C, and clofarabine, and achieved complete remission for the second time. He received a haploidentical (70% matched) bone marrow transplant (BMT) from his son on 03 March 2010, following conditioning with fludarabine, melphalan, and thiotepa, at the MD Anderson Cancer Center. GVHD prophylaxis was tacrolimus 1 mg p.o. twice daily and mycophenolate mofetil 1000 mg p.o. daily. 

Stem cell infusion was uneventful, except for mild hypotension. The patient had mild veno-occlusive disease of the liver that eventually resolved, and several (4-5) episodes of CMV viremia, but no evident GVHD throughout his course. His blood type was A–, but the donor (son) was A+. The patient underwent bone marrow biopsy 3 months after transplantation, which showed 1% blasts and no flow cytometric evidence of relapse. Post-transplant microsatellite polymorphism was compatible with successful engraftment. No chimerism was observed. On post-transplant day 208 he presented with septic/hypotensive shock, and was supported with broad-spectrum antibiotics and intravenous fluids. He did not require mechanical ventilation or vasopressor therapy; however, deep anemia was noted (Hb: 3.8 g dL^–1^) and most of the RBC units cross-matched for transfusion appeared to be incompatible. It was noted that the patient’s blood group had converted to A+, suggesting full erythroid donor chimerism. AIHA was diag nosed, based on fulfillment of all of the following criteria: positive direct antiglobulin test (DAT), negative indirect antiglobulin test (IAT), clinical and laboratory evidence of hemolysis (Table 1), and exclusion of other causes of immune hemolytic anemia. 

Methylprednisolone 1 mg kg–1 (60 mg d–1) IV was initiated. Plasmapheresis with fresh frozen plasma (FFP) was performed (median exchange volume: 3014 mL) for 4 days. After the 1^st^, 2^nd^, and 3^rd^ plasmapheresis, DAT was positive and IAT was negative (Table 1). The reactives for the DAT test were polyspesific for IgG and C3d. Unfortunately, the patient’s Hb value fell to 3.2 g dL–1 at that time (Table 1). IV immunoglobulin (IVIg) 25 g d^–1^ was administered the same day as the 2nd and 3rd plasmapheresis (after apheresis). Methylprednisolone was tapered to 40 mg d–1 on the fifth treatment day. As a result, the patient received 17 units of RBC, plasmapheresis 4 times, and IVIg twice during the course of treatment. Two weeks later AIHA was in control without RBC transfusion and the patient was discharged with an Hb value of 8.5 g dL–1 (Table 1). Methylprednisolone was tapered off within a month. Written informed consent was obtained from the patient. 

Sanz et al. reported that most patients receive steroids as a primary treatment for AIHA and that the majority of cases do not respond [1]. Based on the response in the presented patient, plasmapheresis in addition to IVIg and corticosteroid should be considered a viable alternative treatment option in patients with AIHA that develops during the post-transplant period. 

**Conflict of Interest Statement **

The authors have no conflicts of interest, including specific financial interests, relationships, and/or affiliations, relevant to the subject matter or materials included.

## Figures and Tables

**Table 1:**
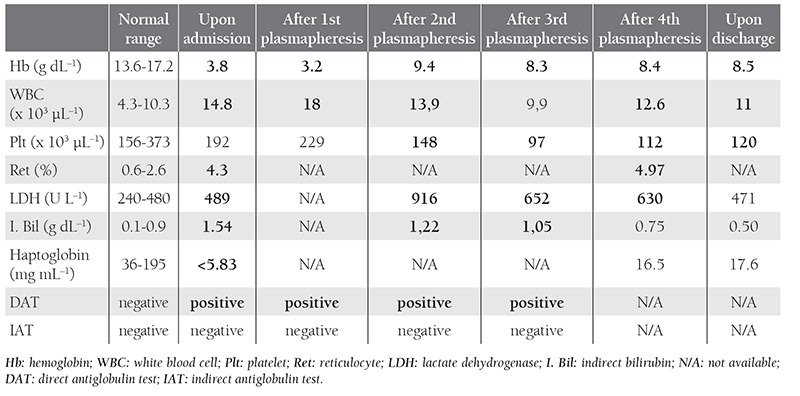
The patient’s laboratory results upon admission, after each plasmapheresis, and upon discharge.
